# Metabolic engineering of potato tuber carotenoids through tuber-specific silencing of lycopene epsilon cyclase

**DOI:** 10.1186/1471-2229-6-13

**Published:** 2006-06-26

**Authors:** Gianfranco Diretto, Raffaela Tavazza, Ralf Welsch, Daniele Pizzichini, Fabienne Mourgues, Velia Papacchioli, Peter Beyer, Giovanni Giuliano

**Affiliations:** 1 ENEA, Casaccia Research Center, PO Box 2400, Roma 00100AD, Italy; 2 Center for Applied Biosciences, Universität Freiburg, Schänzlestrasse 1, 79104 Freiburg, Germany

## Abstract

**Background:**

Potato is a major staple food, and modification of its provitamin content is a possible means for alleviating nutritional deficiencies. beta-carotene is the main dietary precursor of vitamin A. Potato tubers contain low levels of carotenoids, composed mainly of the xanthophylls lutein, antheraxanthin, violaxanthin, and of xanthophyll esters. None of these carotenoids have provitamin A activity.

**Results:**

We silenced the first dedicated step in the beta-epsilon- branch of carotenoid biosynthesis, lycopene epsilon cyclase (*LCY-e*), by introducing, via *Agrobacterium*-mediated transformation, an antisense fragment of this gene under the control of the patatin promoter. Real Time measurements confirmed the tuber-specific silencing of *Lcy-e*. Antisense tubers showed significant increases in beta-beta-carotenoid levels, with beta-carotene showing the maximum increase (up to 14-fold). Total carotenoids increased up to 2.5-fold. These changes were not accompanied by a decrease in lutein, suggesting that LCY-e is not rate-limiting for lutein accumulation. Tuber-specific changes in expression of several genes in the pathway were observed.

**Conclusion:**

The data suggest that epsilon-cyclization of lycopene is a key regulatory step in potato tuber carotenogenesis. Upon tuber-specific silencing of the corresponding gene, beta-beta-carotenoid and total carotenoid levels are increased, and expression of several other genes in the pathway is modified.

## Background

Potato is the fourth most important source of calories for mankind after the cereals wheat, rice and maize. Potato production worldwide stands at 293 million tons, of which 36% in developing countries, and covers more than 18 million hectares [1]. Therefore, the nutritional improvement of potato is of paramount importance to help eradicate nutritional deficiencies.

Vitamin A deficiency affects several hundred million people worldwide [2]. Vitamin A is ingested by humans in two forms: preformed vitamin A (retinol), found in foods of animal origin, and β-carotene (provitamin A), found in dark green vegetables like spinach, in palm oil, and in orange vegetables like yams or carrots.

**Figure 1:**
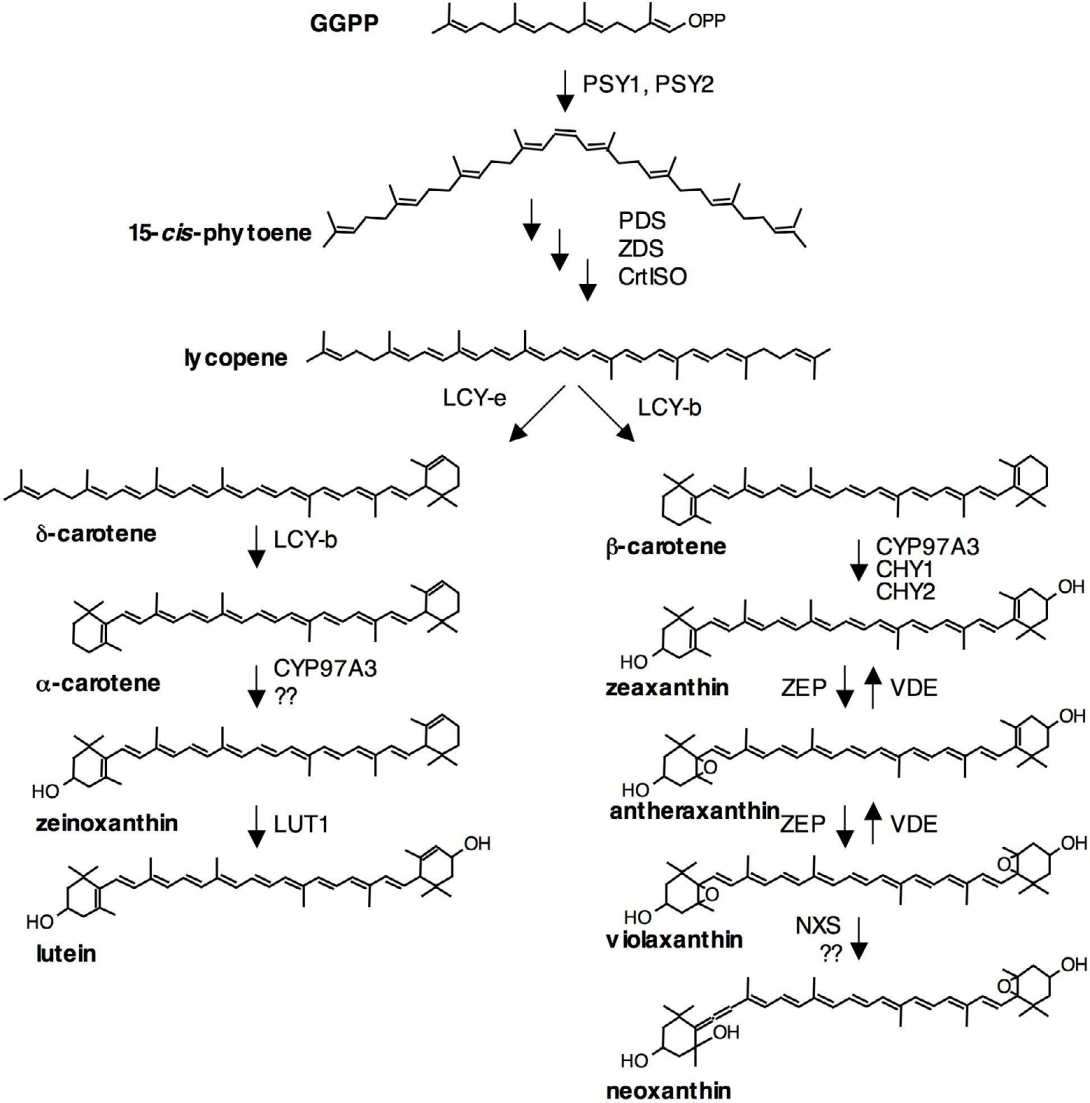
**Carotenoid biosynthesis pathway. **The enzymes involved in the various reactions are indicated.

The first dedicated step in the biosynthesis of carotenoids is the synthesis of phytoene, a colorless compound which is desaturated and isomerized into *all-trans *lycopene (Figure 1). Lycopene is the substrate for two competing cyclases: epsilon-cyclase, introducing a single epsilon-ring, and beta-cyclase introducing one or two beta-rings. Successive hydroxylation and epoxidation reactions result in the synthesis of lutein, on the branch starting at ε-cyclase, and β-β-ring xanthophylls on the branch starting at β-cyclase.

Metabolic engineering of plant carotenoids has been achieved through different strategies (reviewed in [3,4]). These can be summarized in:

i) "push strategies", in which a gene encoding a rate-limiting step in the pathway is overexpressed. Examples of "push" strategies are the overexpression of the *PSY *gene, encoding phytoene synthase, in rice [5], canola [6], tomato [7] and potato [8], of later genes in the pathway, such as those encoding lycopene β-cyclases [9,10] or of "mini-pathways" composed of more than one gene, directing the formation of β-carotene in rice endosperm [11] or of zeaxanthin in tomato fruits [12]. A variant of "push" strategies consists in the introduction of non-plant genes to redirect metabolite flux and synthesize new compounds [13-15].

ii) "block" strategies, relying on the silencing of a biosynthetic step situated immediately downstream of the compound whose levels are to be increased. Examples of "block" strategies are the increase in lycopene in tomato fruits obtained through the silencing of lycopene β-cyclases [9,10], or the increase of zeaxanthin in potato tubers obtained through the silencing of zeaxanthin epoxidase [16]. In this paper, we present a variant "block" strategy, aimed at redirecting the metabolic flux into the β-β branch of the bifurcated carotenoid pathway by silencing the first step of the competing ε-β branch. We present the phenotypic analysis of transgenic potato plants in which the first biosynthetic step in the branch leading to lutein, lycopene ε-cyclase (Figure 1), has been inhibited through a tuber-specific antisense approach.

## Results

### Construction of transgenic plants

**Figure 2:**
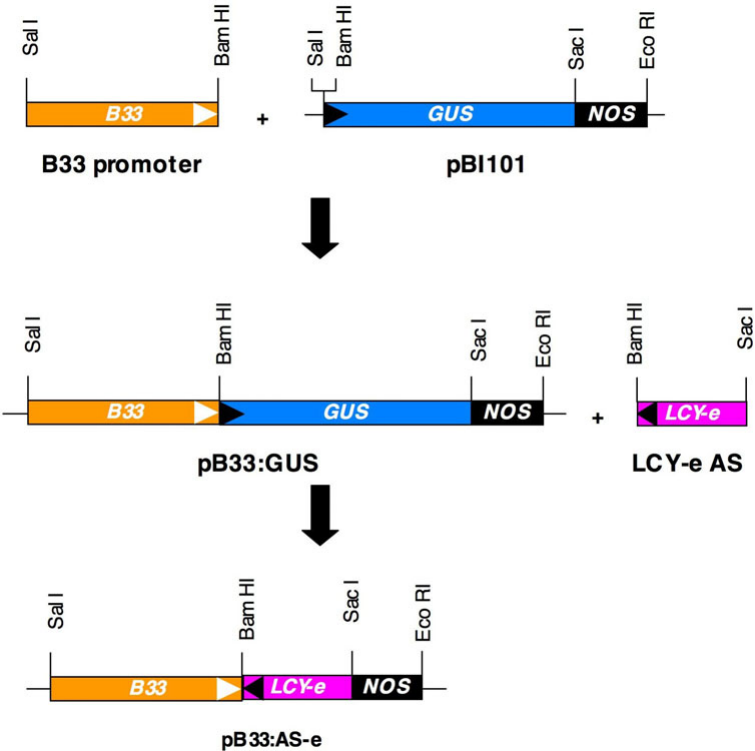
**Construction of plasmids for *Agrobacterium*-mediated transformation. **The patatin *B33 *promoter and a region of the *LCY-e *transcript were amplified, respectively, from genomic DNA and tuber cDNA using primers inserting appropriate restriction sites at the ends, resequenced, and cloned in the pBI101 plasmid [21]. White triangles indicate the direction of transcription, black triangles the direction of the coding strand. For details see Methods.

The first dedicated step in lutein biosynthesis is the ε-cyclization of lycopene by LCY-e (Figure 1). In cv. *Desirée*, lutein constitutes about 12% of tuber and 48% of leaf carotenoids, respectively. In Arabidopsis, *lut2 *mutants, which are deficient in ε-cyclization, are viable [17] but, under stress conditions, show reduced chlorophyll triplet quenching and, therefore, enhanced photooxidation [18]. In order not to interfere with leaf lutein biosynthesis, we decided to silence the lycopene ε-cyclase transcript *(LCY-e*) specifically in tubers. Several examples of organ-specific silencing are known in plants, such as the fruit-specific silencing of *LYCOPENE *β-*CYCLASE *(*LCY-b*) [9] or of the *HP-2 *gene [19] in tomato. In the present work, the patatin promoter [20] was used to target the expression of the silencing transcript in tubers. Appropriate fragments of the patatin *B33 *promoter [20] and of the *LCY-e *transcript were amplified by PCR, re-sequenced, and cloned in the *pBI101 *vector for *Agrobacterium*-mediated transformation [21] (see methods). Two plasmids were constructed: plasmid *pB33:GUS *contains the *GUS *reporter gene under the control of the *B33 *promoter, while plasmid *pBI33:AS-e *contains an antisense *LCY-e *cDNA fragment replacing *GUS *(See Figure 2 and Methods).

The two constructs were introduced in potato (*cv Desirée*) using published methods [22] and transgenic plants were selected on kanamycin. The presence of the transgene was confirmed via PCR (see Methods). Two independent transgenic lines for *GUS *and 6 for *AS-e *were selected for further characterization. For each clone, two plants were acclimated in the greenhouse for tuberization. At the end of the life cycle, tubers were harvested and the tuber production was evaluated. Neither *GUS *nor *AS-e *transgenic plants showed major alterations in tuber weight or number (data not shown).

### Tuber and leaf carotenoid composition

**Figure 3:**
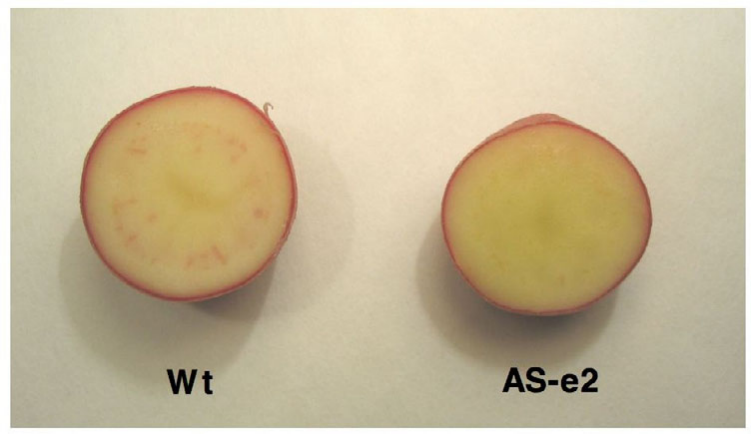
**Pigmentation of Wt and transgenic tubers. **Tubers were photographed immediately after harvesting.

The carotenoid composition of tubers from the *Wt*, one of the *GUS *lines and all the *AS-e *lines was analyzed through diode array HPLC (Table 1). The *GUS *line did not show significant changes in carotenoid content, with respect to wild-type *Desirée*. In contrast, four out of the six *AS-e *lines (lines 1 to 4) showed significant (>1.8-fold) increases in total tuber carotenoids, as well as changes in carotenoid composition. This increase was reflected in a minor increase in yellow pigmentation of the tubers (Figure 3). Consistent with the hypothesized silencing of *LCY-e*, the levels of β-β-carotenoids (β-carotene, zeaxanthin, antheraxanthin, violaxanthin and neoxanthin, see Figure 1) showed significant increases (up to 3-fold) in these lines. β-carotene was the single carotenoid compound showing the highest relative increases, up to 14-fold in line *AS-e 4*. Somewhat surprisingly, the levels of lutein remained fairly constant in most of the *AS-e *lines. The ratio of β-β- to β-ε-carotenoids increased from 5.4 in the wild-type, up to 16.3 in line *AS-e 2*.

A slight increase of the colorless biosynthetic intermediate, phytofluene, was observed in lines *AS-e *1 to 4. The other early intermediates (phytoene, ζ- carotene) were below detection in all lines.

These changes in carotenoid content must be attributed to a specific effect of the introduced *AS-e *transgene, and not to non-specific events occurring during plant regeneration, such as somaclonal variation. These non-specific events account for a very limited variation in carotenoid levels, as shown by the data relative to the *GUS *line (Table 1) which shows only minor changes with respect to the wild-type.

Consistently with the tuber-specific nature of the promoter used for the silencing construct, no significant variations in carotenoid or chlorophyll content were observed in leaves of *GUS *or *AS-e *lines (data not shown).

### Transgene expression in tubers and leaves

**Figure 4:**
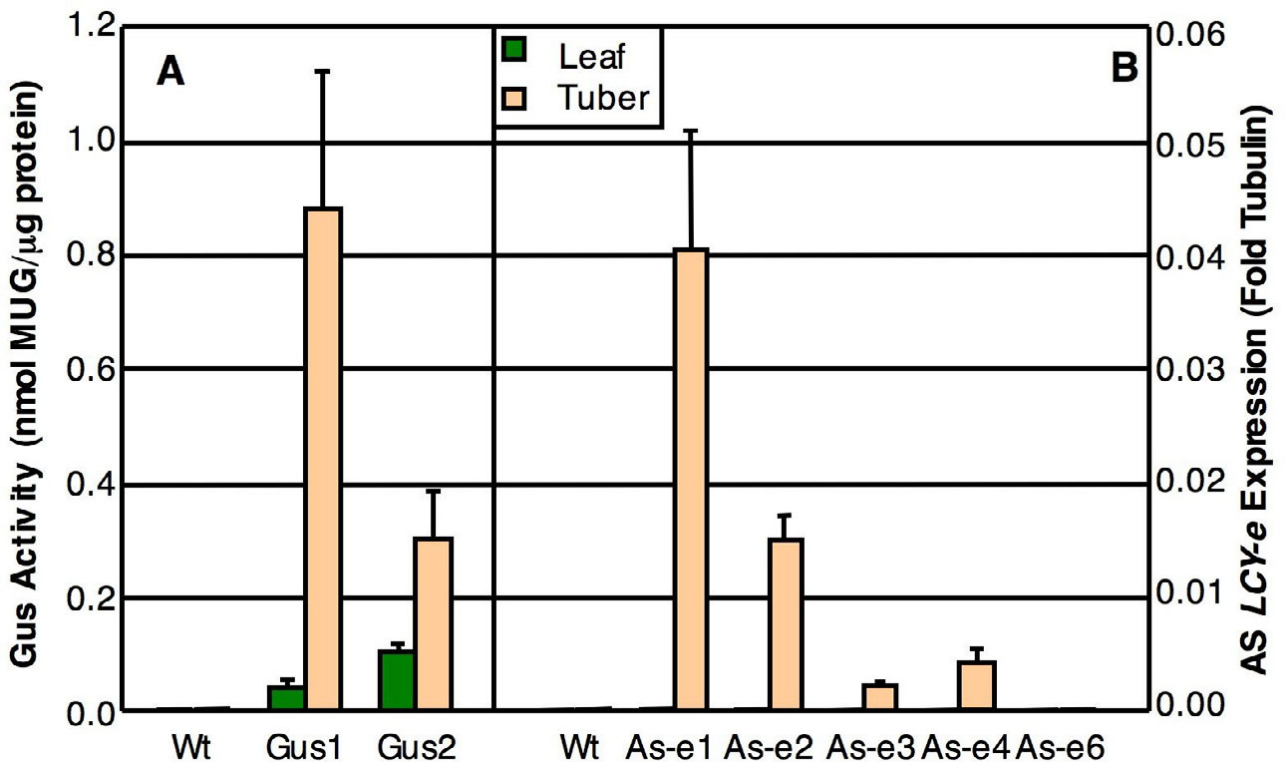
**Transgene expression. **(A): GUS activity was measured fluorimetrically. (B): *AS-e* tranSgene expression was measured via Real Time RT-PCR. For details see Methods.

We performed fluorimetric GUS assays on the *GUS *lines and Real Time RT-PCR assays on four *AS-e *lines showing significant variations in tuber carotenoids (*AS-e 1 *to *4*) and one showing wild-type carotenoid composition (*AS-e 6*). The results are shown in Figure 4. GUS activity was found in tissues of *GUS *transgenic plants, but not *Wt *plants, and was preferentially expressed in tubers. The *AS-e *silencing transcript was detected in the tubers of all the *AS-e *lines, except for *AS-e 6*. Lack of expression of the *AS-e *transcript in this line is not due to improper transgene integration, since the transgene is present and is not rearranged (data not shown). The silencing transcript was below the levels of detection of the Real Time assay in the leaves of all the *AS-e *lines (Figure 4). The levels of expression of the transgene in the tubers were rather low, ranging from 0.04-fold to 0.002-fold β-tubulin. Other than the lack of transgene expression in line *AS-e 6*, no evident correlation between the levels of transgene expression and carotenoid composition of tubers (Table 1) were observed.

### Perturbations in endogenous carotenoid gene expression

Expression of some genes in the carotenoid pathway can be forward- or feedback-regulated by changes of the levels of pathway intermediates: for instance, the *PDS *transcript, encoding phytoene desaturase, is induced in leaves accumulating phytoene as a result of the *ghost *mutation or after treatment with the herbicide norflurazon [23,24], suggesting that expression of this gene may be driven by the concentration of the substrate of the reaction. In agreement with this hypothesis, tubers overexpressing a bacterial phytoene synthase show increased levels of the *PDS *transcript [8].

**Figure 5:**
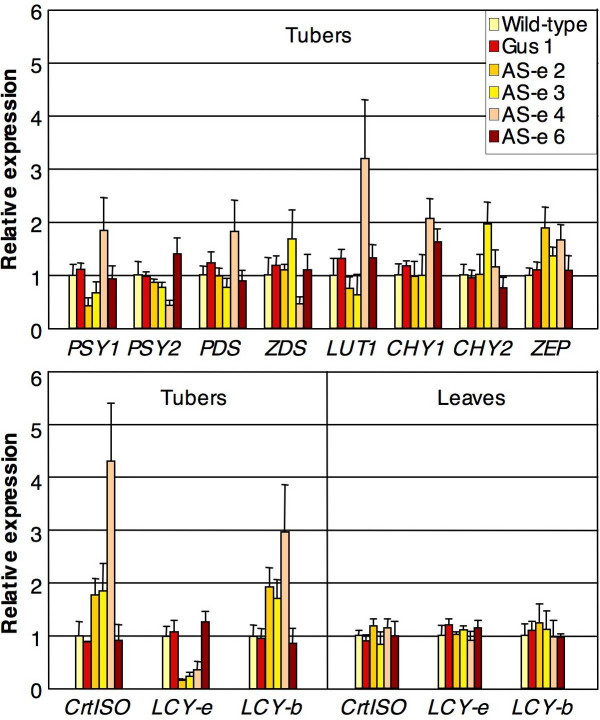
**Endogenous carotenoid gene expression. **Transcript levels were measured through Real Time RT-PCR and were first normalized for expression of the housekeeping β-tubulin gene, and then for the expression levels in the Wt. Data show the average and SE (error bars) of determinations from at least 4 different tubers (or leaves) from 2 different plants. For details see Methods.

We measured the expression of carotenoid gene transcripts in the tubers, using Real Time quantitative RT-PCR. Alongside the *Wt*, we analyzed lines *GUS1 *and *AS-e6*, showing wild-type carotenoid levels, and lines *AS-e 2*, *3 *and *4*, showing significant changes in carotenoid composition. We analyzed transcripts for most genes in the carotenoid pathway (*PSY1*, *PSY2*, *PDS*, *ZDS, CrtISO*, *LCY-b, LCY-e, CHY1, CHY2, LUT1*, *ZEP*). The biosynthetic steps catalyzed by these genes are shown in Figure 1. The identity of the transcripts used for designing the oligonucleotides used for RT-PCR, is indicated in Methods. The tuber transcript levels, normalized first for the β-tubulin transcript and then for the Wt, are shown in Figure 5.

The *GUS1 *line, as well as line *AS-e6*, showed only minor variations in gene expression with respect to the *Wt *line. This indicates that *in vitro *culture conditions, somaclonal effects due to regeneration procedures, or the presence of the silencing transgene by itself, do not cause any major variability in endogenous carotenoid gene expression. The endogenous *LCY-e *transcript shows consistent silencing in *AS-e *lines *2,3 *and *4*, but not in the other lines. Line *AS-e 2*, which is the one showing the highest increase in β-β-xanthophylls (Table 1), also shows the most efficient silencing of endogenous *LCY-e*.

Some clear trends in gene expression can be identified: *CrtISO*, *LCY-b *and *ZEP *show an increase in all the *AS-e *lines, with the exception of line *AS-e 6*, which has a wild-type carotenoid content. Other transcripts show more variable trends, being induced or repressed in only one or two of the *AS-e *lines.

Line *AS-e4*, which is the one showing the highest increase in total carotenoid levels (Table 1), stands out from the other lines analyzed, in that it shows a simultaneous induction of several transcripts: *PSY1, PDS, CrtISO, LCY-b, LUT1, and CHY1*.

The transcript levels of the three genes showing significant changes in tubers (*LCY-e, Lcy-b and CrtISO*) were measured also in leaves (Figure 5). In this tissue, they showed only minor oscillations. This confirms that, similarly to the alterations in carotenoid content, also the perturbations in endogenous carotenoid transcript levels were limited to tubers, the target tissue of *AS-e *transgene overexpression.

## Discussion

Like for any other metabolic pathway, flux through the carotenoid pathway is thought to be controlled by rate-limiting enzymatic steps, whose modification through metabolic engineering should be able to change carotenoid composition. Metabolic flux analysis [25] should allow the identification of these rate-limiting steps, and predict the effects of their engineering. However, the experimental verification of these predictions often contradicts theory, mainly because the levels of expression of some of the genes, and thus of the corresponding enzymes, are sensitive to the levels of the intermediates and of the final products of the pathway.

In potato, overexpression of early genes of the carotenoid pathway is a promising strategy for increasing total carotenoids and β-carotene in the tuber. Overexpression of the bacterial *CrtB *gene, encoding phytoene synthase, leads to up to 7-fold increase in total carotenoids, mainly β-carotene and lutein [8]. This indicates that PSY is a rate-limiting step in wild-type tubers.

The role of LCY-e and LCY-b in directing the flux towards the synthesis of β-β and β-ε carotenoids can hardly be underestimated. Modulating the levels of these two competing cyclases is a strategy for obtaining tubers with a defined carotenoid composition. The results presented here indicate that the *LCY-e *transcript can be silenced in a tuber-specific fashion, resulting in a tuber-specific increase of β-β-carotenoids (up to 3-fold), and, more specifically, of β- carotene (up to 14-fold).

Real Time RT-PCR measurements showed that the silencing transgene, introduced under the control of the patatin promoter, was expressed in a tuber-specific fashion in all but one of the transgenic lines. The transgene was expressed at rather low levels, which is not surprising, since homology-driven post-transcriptional gene silencing is known to be a bidirectional phenomenon, affecting both the target gene and the silencing construct [26]. The lack of expression of the transgene in line *AS-e 6 *awaits an explanation, since the construct is stably integrated in the genome and it is not rearranged (data not shown). Whatever the case, this line, together with the *GUS1 *line, shows wild-type carotenoid composition, reinforcing the hypothesis that the alterations in carotenoid content observed in lines *AS-e 1 *to *4 *are indeed the effect of expression of the *AS-e *transgene, and thus of the silencing of endogenous *LCY-e*. In fact, in all the lines expressing the *AS-e *transgene, the endogenous *Lcy-e *transcript was silenced, to various extents. The silencing was confined to tubers. This result suggests that, similarly to tomato fruits [9,19], a physical barrier must exist, preventing the spreading of the silencing signal outside of the tuber tissue.

The tuber-specific changes in *LCY-e *gene expression were accompanied by tuber-specific changes in carotenoid composition: first, total carotenoid levels increased significantly, albeit to variable extents; second, this increase involved, as expected, mostly carotenoids of the β-β- branch; third, β-carotene showed the highest relative increases (up to 14-fold) among all carotenoid compounds. Surprisingly, lutein showed little, if any, reduction in the silenced tubers. This observation suggests that LCY-e activity is not rate-limiting for tuber lutein levels. It is possible that, to decrease lutein, much higher levels of silencing of LCY-e are required, similar to the ones obtained with RNAi constructs [27]. Several additional enzymes, such as carotenoid hydroxylases [28,29] are involved in lutein synthesis. Furthermore, it is also possible that lutein levels are also controlled by hitherto undescribed carotenoid cleavage activities [30].

Endogenous carotenoid gene expression shows two distinct patterns of regulation in silenced tubers: three transcripts (*CrtISO*, *LCY-b *and *ZEP*) are significantly induced in all the silenced lines. This indicates that these transcripts are sensitive either to the levels of *LCY-e *expression, or to the levels of one or more carotenoid pathway intermediates.

In one of the lines, *AS-e 4*, showing the highest total carotenoid and β-carotene levels, a generalized induction of carotenoid transcripts is observed (*CrtISO*, *LCY-b *and *ZEP*, but also *PSY1*, *PDS *and the two paralogs showing preferential expression in tubers, i.e. *LUT1 *and *CHY1*). This result can be interpreted in two different ways:

a) the generalized induction of carotenoid gene transcripts could be an *effect *of the more extreme carotenoid level perturbation observed in this line with respect to the other ones

b) instead, the generalized induction could be the effect of a mutation, or of another somaclonal event, occurred during transformation or regeneration of this line, and having a generalized inductive effect on the carotenoid pathway. In this case, the induction of carotenoid gene expression, added to the silencing of LCY-e, would be the *cause *of the extreme variation in carotenoid content observed in this line.

No major changes in tuber productivity or leaf carotenoid composition were observed in any of the lines, suggesting that silencing of *LCY-e *is a viable strategy for changing tuber carotenoid composition without affecting agronomic performance. Field trials are however needed to verify the agronomic performance of these lines.

The presence of gene paralogs showing preferential expression in tubers constitutes an interesting parallel with tomato, a close relative of potato, where it has been suggested that duplication of the *PSY *and *CHY *genes has created two parallel pathways, one active in leaves and the second active in chromoplast-containing organs (fruits and flowers) [31]. In agreement with this hypothesis, mutation or silencing of *PSY1 *in tomato affects fruit and flower, but not leaf carotenoids [32,33]. It remains to be demonstrated what is the relative contribution of the different members of the *PSY *and *CHY *gene families to potato tuber carotenogenesis.

## Conclusion

Using an antisense construct under the control of the tuber-specific patatin promoter, we obtained the tuber-specific silencing of the potato *LCY-e *transcript. The amount of β-β-carotenoids increased accordingly in a tuber-specific fashion, without a parallel decrease of the major tuber β-ε- carotenoid, lutein. This modification in carotenoid content is paralleled by modifications in endogenous carotenoid gene expression. The strategy described here, in combination with previously described ones, such as the overexpression of phytoene synthase and the antisense silencing of zeaxanthin epoxidase, constitutes a toolbox for the metabolic engineering of the carotenoid content of potato tubers.

## Methods

Unless indicated differently, molecular biology methods are as described in [34]. The cloning strategy is outlined in Figure 2. Briefly, a 1.53 Kb patatin *B33 *promoter fragment [20] was amplified from potato (*cv Berolina*) genomic DNA using the primers B33-up and B33-dw (Table 2). These primers inserted, respectively, Sal I and Bam HI sites upstream and downstream of the promoter sequence. After intermediate cloning in the *pBSK+ *vector and re-sequencing, the fragment was inserted in the *pBI101 *vector [21] upstream of the *GUS *reporter gene, resulting in the plasmid *pBI33:GUS*.

A 0.75 Kb *LCY-e *cDNA fragment was amplified from potato (*cv Desirée*) tuber cDNA using the primers LCY-e 322 up and LCY-e 1085 dw (Table 2). These primers inserted, respectively, Sac I and Bam HI sites upstream and downstream of the cDNA fragment. After intermediate cloning in the *pBSK+ *vector and re-sequencing, the fragment was inserted, in antisense orientation, instead of the *GUS *gene in the *pBI33:GUS *vector, resulting in the plasmid *pBI33: AS-e *(Fig. 2).

Potato (*cv Desirée*) was transformed as previously described [22]. Plantlets growing on kanamycin were tested by PCR, using primers Lcy-e 574 Up and Nos-test 2. PCR-positive, rooted plantlets were adapted in greenhouse in pots (diameter: 25 cm) in a soil mixture composed of 1/3 sand and 2/3 of sterile soil (Terraplant 2, BASF). Photoperiod was set at 14 hours of light and 10 hours of dark, with temperature set at 24°C during the light period and at 16°C during the dark period. In the advanced phase of growth, the day temperature was kept around 20°C in order to promote tuberization. During tuberization, irrigation was reduced in order to prevent tuber decay.

Tubers from the lower 2/3 of the pot ("deep" tubers) were collected separately from superficial ones, washed in water, dried briefly at room temp, cut in pieces and frozen at -80°C. Tuber productivity for each line was estimated as the total number of tubers produced and as their total weight. All carotenoid and RT-PCR measurements were conducted on at least 4 different "deep" tubers per each line, to prevent possible alterations in carotenoid composition/gene expression resulting from light accidentally illuminating the superficial tubers.

Total RNA was isolated from frozen tissue and analyzed through Real Time RT-PCR using previously published methods [23,35]. Two independent RNA extractions and four cDNAs (two from each RNA) were used for the analyses; first strand cDNA was synthesized from 0.5 μg of RNA in 20 μl with oligo-dT(16) and Superscript II (Invitrogen), according to the manufacturer's instructions. QRT-PCR were performed using an ABI PRISM 7000 instrument and the SYBR Green Master Mix kit (cat. 4309155; Applera). Standard dilution curves were performed for each gene fragment and all data were normalized for the level of the *β-TUBULIN *transcript and for wild-type expression levels. Primers for Real Time experiments were designed using the Primer Express v2.0 software and validated with the Amplify v3.1 software. Sequences of the primers used for the various genes are shown in Table 2. The sequences used to design the primers were: *β-TUBULIN*: Z33382; *PSY1*: TC122598; *PSY2*: L23424 (from tomato); *PDS*: AY484445; *ZDS*: TC114158; *CrtISO*: TC117194; *LCY-e*: AF321537; *LUT1*: TC117729; *LCY-b*: X86452 (from tomato); *CHY1*: TC36005; *CHY2*: TC32024; *ZEP*: EST724320 (all numbers refer to GenBank accession numbers, with the exception of those starting with TC, which are Transcript Contig numbers from TIGR potato gene index release 10.0 [36]. In order to discriminate the introduced *AS*-e mRNA from the endogenous *Lcy-e *mRNA, the former was amplified using primers AS-e Up and AS-e Dw, while the latter was amplified using primers Lcy-e Up and Lcy-e Dw.

Fluorimetric GUS assays were performed as described previously [24].

For HPLC analysis, frozen tubers were lyophilized, peeled, ground to powder and pigments were extracted three times with 2 ml of acetone. In the first extraction, 200 μg tocopherol acetate per sample was added as an internal standard. Combined acetone extracts were dried, lipophilic compounds were resuspended in 2 ml of petroleum ether:diethyl ether (2:1, v/v) and 1 ml of distilled water. After centrifugation for 5 min at 3000 × g the organic phase was recovered and the aqueous phase was extracted for a second time as described above. The combined organic phases were dried and dissolved in 30 μl chloroform. 10 μl were subjected to quantitative analysis using HPLC with a C30 reversed-phase column (YMC Europe GmbH, Schermbeck, Germany) and a gradient system as described [37]. Carotenoids were identified by their absorption spectra, monitored using a photodiode array detector (PDA 2996; Waters, Eschborn, Germany). Normalization of the samples to the internal standard and the quantification of carotenoid amounts was performed as described previously [38].

## Authors' contributions

GG, RT and PB planned and supervised the work. FM prepared the constructs for transformation, RT and VP performed the transformations and maintained the lines in vitro, DP and GD grew and sampled the plants, DP performed the GUS assays and GD the Real Time assays, RW performed the HPLC's.

## Figures and Tables

**Table 1:** HPLC analysis of tuber carotenoids (ng/g dry weight). Carotenoids were measured via diode array HPLC (see Methods) on a minimum of 4 different tubers from 2 different plants. Fold variation with respect to the wild-type is reported for each carotenoid compound and for each line.

**Line**	**Phytofluene**	**Lutein**	**β-carotene**	**Zeaxanthin**	**Antheraxanthin**	**Violaxanthin**	**Neoxanthin**	**Other Xanthophylls**	**Esters**	**Total Carotenoids**
**Wild-type**	43.07± 12.78	588.01± 108.14	3.17± 1.01	262.64± 108.08	1301.33± 479.34	1109.08± 420.06	541.02± 98.14	89.34± 13.02	734.35± 173.04	4672.02± 1414.62
**Gus 1**	52.01± 5.87	531.46± 153.57	3.76± 1.32	223.02± 63.04	1091.14± 293.24	1157.69± 332.49	685.32± 204.95	81.34± 22.40	1270.39± 299.42	5096.15± 1376.25
**Fold Variation**	**1.21**	**0.90**	**1.20**	**0.85**	**0.84**	**1.04**	**1.27**	**0.91**	**1.73**	**1.090**
**AS-e 1**	160.59± 8.03	725.46± 100.33	17.17± 3.59	626.28± 187.37	2173.42± 97.33	2788.91± 170.82	303.66± 83.88	110.94± 38.43	2015.11± 525.48	8921.56± 1218.27
**Fold Variation**	**3.73**	**1.23**	**5.42**	**2.38**	**1.67**	**2.51**	**0.56**	**1.24**	**2.74**	**1.909**
**AS-e 2**	248.59± 62.92	506.12± 112.22	14.35± 3.14	669.39± 50.98	3045.66± 493.89	3320.07± 1061.82	1219.54± 20.21	158.88± 79.35	3089.75± 69.24	12272.36± 1961.77
**Fold Variation**	**5.77**	**0.86**	**4.53**	**2.55**	**2.34**	**2.99**	**2.25**	**1.78**	**4.21**	**2.627**
**AS-e 3**	86.97± 6.34	866.91± 104.46	9.48± 1.84	465.48± 146.89	1703.35± 150.12	2917.70± 720.57	945.84± 318.98	125.80± 55.78	1555.41± 589.078	8677.12± 2094.07
**Fold Variation**	**2.02**	**1.47**	**2.99**	**1.77**	**1.31**	**2.63**	**1.75**	**1.41**	**2.19**	**1.857**
**AS-e 4**	193.70± 1.23	1044.59± 144.40	43.56± 14.24	990.83± 197.51	2198.54± 559.12	2935.03± 797.13	612.56± 245.85	90.14± 24.62	1865.33± 1010.02	9974.29± 2994.11
**Fold Variation**	**4.50**	**1.78**	**13.74**	**3.77**	**1.70**	**2.65**	**1.13**	**1.01**	**2.54**	**2.135**
**AS-e 5**	35.44± 5.22	510.42± 99.42	2.60± 0.92	324.83± 111.81	1266.71± 78.11	1124.44± 409.81	421.81± 173.67	98.73± 20.30	776.25± 87.11	4561.23± 986.38
**Fold Variation**	**0.82**	**0.87**	**0.82**	**1.24**	**0.97**	**1.01**	**0.78**	**1.10**	**1.06**	**0.977**
**AS-e 6**	50.21± 8.87	469.53± 137.01	3.32± 2.01	338.81± 87.43	1369.53± 263.04	1378.81± 359.37	746.48± 221.87	76.81± 13.37	688.91± 118.44	5123.42± 1211.42
**Fold Variation**	**1.17**	**0.80**	**1.05**	**1.29**	**1.05**	**1.24**	**1.38**	**0.86**	**0.99**	**1.097**

**Table 2:** Primers used. Sequences of the primers used for cloning of the promoter and gene fragments, for PCR screening of the putative transgenic plants, and for Real Time quantification of transcript levels. For further details, see Methods.

**Primer name**	**Sequence**	**Use**
B33-149 Up	ACATATTTTACTTTTGACTTT	B33 cloning
B33-1950 Dw	GTAGTGTCACACTTACGC	B33 cloning
B33 Up	AGTCGACAATCATTGTTTTATTTTCTCTTTC	B33 cloning
B33 Dw	AGGATCCAAATTTTGTTGGTGCTTTGAG	B33 cloning
Lcy-e 322 Up	AGAGCTCGGCTGTGGTCCTGCTG	Lcy-e cloning
Lcy-e 1085 Dw	AGGATCCTGGCAAAGATCCACCAAC	Lcy-e cloning
Lcy-e 574 Up	TAGGATCATCATCATCAAG	Pcr screening
Nos-test2	CGCGTATTAAATGTATAATTG	Pcr screening
Tub 865 Up	CTGTGAATTTAATCCCCTTG	Pcr screening
Tub 1260 Dw	TGCGCCTGAACATGGCAGT	Pcr screening
Tub Up	CAGACCTGAGGAAATTGGCTG	Real Time assay
Tub Dw	TTCTTGGCATCCCACATTTGT	Real Time assay
Ubi Up	ACAATGTCAAAGCCAAGATCCA	Real Time assay
Ubi Dw	CGGAGACGGAGCACGAGA	Real Time assay
AS-e Up	CCCCAATTTAGCAGACTCCG	Real Time assay
AS-e Dw	CAACAGAAATTATATGATAATCATCGCAA	Real Time assay
Psy1 Up	CGGTCTGCTATTGTTGCTACTCC	Real Time assay
Psy1 Dw	CAGGAACAGGTATGTCTGGCTTC	Real Time assay
Psy2 Up	AGCTTTAGATAGGTGGGAGGCA	Real Time assay
Psy2 Dw	CAAGTCCATACGCATTCCTTCAA	Real Time assay
PDS Up	AGAGACTTTGCATGCCGATTGT	Real Time assay
PDS Dw	AAAGCATCGCCCTCAACTGT	Real Time assay
ZDS Up	TTGCCATGTCAAAGGCCA	Real Time assay
ZDS Dw	ACAGGCACTCCGACCAATTT	Real Time assay
CrtIso Up	TTGGCAGCAGTAGGACGTAAAC	Real Time assay
CrtIso Dw	TCCCTTCCTTTTCATGTGGAA	Real Time assay
Lcy-e Up	GCCAAAATGGATGTGGCAG	Real Time assay
Lcy-e Dw	CAATGTTGCACCAGTAGGATCAG	Real Time assay
Lut1 Up	CGTTCTCCGCCCAAAAAAC	Real Time assay
Lut1 Dw	TTGGCCTAAAGTAAGTGACCTGG	Real Time assay
Lcy-b Up	AATGGGTGGTCCACTTCCAGTA	Real Time assay
Lcy-b Dw	GGATGGATGAACCATGCCAG	Real Time assay
Chy1 Up	CTTGGCCCAAAACCCACTT	Real Time assay
Chy1 Dw	CCTCAAATTGAGGTTTCAGCTTCT	Real Time assay
Chy2 Up	TTTTGCTGTCTCGAAGAAAGCC	Real Time assay
Chy2 Dw	AGCCAACAGGCAGCTAAACTCT	Real Time assay
ZEP Up	TCATGAATGCTGGCTGCATC	Real Time assay
ZEP Dw	TGCTGCAAAGTCATGCGG	Real Time assay
